# Accurate Co-Localization of Luciferase Expression and Fluorescent Anti-CEA Antibody Targeting of Liver Metastases in an Orthotopic Mouse Model of Colon Cancer

**DOI:** 10.3390/cancers16193341

**Published:** 2024-09-29

**Authors:** Kyung-Ha Lee, Kristin E. Cox, Siamak Amirfakhri, Sunidhi Jaiswal, Shanglei Liu, Mojgan Hosseini, Thinzar M. Lwin, Paul J. Yazaki, Robert M. Hoffman, Michael Bouvet

**Affiliations:** 1 Department of Surgery, University of California San Diego, La Jolla, CA 92037, USA; kyl031@ucsd.edu (K.-H.L.); rhoffman@ucsd.edu (R.M.H.); 2 VA San Diego Healthcare System, La Jolla, CA 92161, USA; 3 Department of Colorectal Surgery, Chungnam National University Hospital, Daejeon 35015, Republic of Korea; 4 Department of Pathology, University of California San Diego, La Jolla, CA 92037, USA; 5 Department of Surgical Oncology, City of Hope, Duarte, CA 91010, USA; 6 Department of Immunology and Theranostics, Beckman Research Institute, City of Hope, Duarte, CA 91010, USA; 7 AntiCancer Inc., San Diego, CA 92111, USA

**Keywords:** fluorescence-guided surgery, tumor labeling, bioluminescence, colorectal cancer, liver metastases, M5A, orthotopic nude mouse model

## Abstract

**Simple Summary:**

The present study demonstrated that a humanized anti-CEA antibody conjugated to a near-infrared dye accurately co-localized with luciferase expressing colorectal cancer liver metastases in an orthotopic mouse model. The present study validates the metastatic tumor targeting specificity of the anti-CEA antibody.

**Abstract:**

Background: The present study aimed to validate the accuracy of a tumor-specific antibody to target liver metastases of colorectal cancer. Methods: A humanized anti-CEA antibody conjugated to a fluorescent dye (M5A-IR800) was tested for targeting human colorectal cancer liver metastases (CRLMs) expressing luciferase in an orthotopic mouse model. Orthotopic mouse models of CRLMs were established by implanting fragments of a luciferase-expressing human colorectal cancer cell line, LS174T, in the liver of nude mice. Mice received 50 µg M5A-IR800 72 h prior to imaging. To test co-localization, bioluminescence imaging was performed using D-luciferin, which was given via intraperitoneal injection just prior to imaging. Results: Tumors were able to be visualized non-invasively through the skin with the luciferase–luciferin signal. Intra-abdominal imaging showed accurate labeling of CRLMs with M5A-IR800, which co-localized with the luciferase–luciferin signal. Conclusions: The present results validate the accuracy of a tumor-specific anti-CEA antibody in targeting liver metastases of colorectal cancer.

## 1. Introduction

For the treatment of metastatic colorectal cancer, complete resection of metastases is the standard strategy, which can increase clinical remission rates and survival rates. The liver is the most common metastatic site for colorectal cancer, and it is reported that approximately 30–50% of all patients will eventually develop liver metastases [1]. The survival of patients with colorectal liver metastases (CRLMs) has markedly improved, with a 5-year overall survival rate of 25–40%, due to improvements in imaging modalities, surgical techniques, systemic chemotherapy, local treatment, and multidisciplinary tumor board management [2]. However, complete surgical resection of CRLMs is the key element to achieve remission and improve prognosis [3]. Nevertheless, the intraoperative detection of liver metastases can be challenging in many cases as smaller metastases can be difficult to detect by the naked eye or even by intraoperative ultrasonography. Additionally, some benign liver lesions can be mistaken for metastases.

As a surgical navigation technology, fluorescence-guided surgery (FGS) has become a promising tool for the intraoperative detection of many cancers, including CRLMs [4,5,6,7,8,9,10,11,12]. Additionally, FGS can be used for the identification of anatomy or confirmation of perfusion [13,14]. Recently, preclinical studies have introduced tumor-specific antibodies for FGS and demonstrated their efficacy in animal models. Tumor-specific FGS for CRLMs could improve the rate of complete resection and allow the detection of occult lesions [15].

A humanized anti-carcinoembryonic antigen (CEA) antibody conjugated to a near-infrared 800 nm dye (M5A-IR800) has been shown to target tumors with CEA expression [16,17]. We have previously reported that M5A-IR800 brightly targeted primary colorectal cancer and CRLMs in an orthotopic cell line model [18]. This prior experiment established proof of principle that M5A-IR800 could detect CRLMs. The present study aimed to demonstrate the co-localization of luciferase expression from the CRLM cells and M5A-IR800 targeting in an orthotopic nude mouse model of CRLMs to validate the accuracy of a tumor-specific antibody to visualize liver metastases as the last major step before clinical use in tumor staging and resection.

## 2. Materials and Methods

### 2.1. Mice

Athymic male and female nude mice, 4–6 weeks of age, purchased from the Jackson Laboratory (Bar Harbor, ME, USA) were used for this study. Mice were housed in a biosafety room and fed an autoclave diet. Prior to any surgical procedure, mice were anesthetized with an intraperitoneal injection of 20–25 µL of a solution of xylazine and ketamine, reconstituted in phosphate-buffered saline (PBS). They received a subcutaneous injection of buprenorphine reconstituted in PBS (dosage: 0.05 mg/kg) for postoperative pain control. At the end of the study, mice were anesthetized with isoflurane inhalation and euthanized by cervical dislocation. All studies were approved by the San Diego Veterans Administration Medical Center Institutional Animal Care and Use Committee (IACUC) Animal Use Protocol A17-020 and the University of California San Diego (UCSD) IACUC Protocol S99001.

### 2.2. Establishment of Orthotopic CRLM Mouse Models

The human colon cancer cell line LS174T (American Type Culture Collection, Manassas, VA, USA) was used for this study. Stably transduced LS174T cells expressing the firefly luciferase gene were generated by lentiviral transfection of the pGL4 Luciferase Reporter Vector (Promega, Fitchburg, WI, USA). To prepare tumors for implantation, subcutaneous tumor models were established by injecting LS174T–luciferase cells (1 × 10^6^) reconstituted in 100 µL of media (Corning Life Sciences, Corning, NY, USA) into the bilateral flanks of nude mice. When tumor size reached at least 5 mm^3^, mice were euthanized and the tumors were excised and cut into 1 mm^3^ fragments. For orthotopic implantation, a sutureless surgical orthotopic implantation liver technique was used as previously described [19]. In brief, the abdomen of the mouse was sterilized with a 70% ethanol solution and an approximately 10 mm vertical incision was made at the upper midline of the abdomen. The left lobe of the liver was carefully extracted extracorporeally and a 2 mm incision was made through the liver parenchyma with scissors. A tumor fragment was placed inside the liver through the incision and the implantation site was gently compressed to achieve hemostasis. The left lobe of the liver was carefully returned to the abdomen and the incision was closed with interrupted 6-0 nylon sutures (Ethicon Inc., Somerville, NJ, USA). Orthotopic mouse models were allowed to grow for 2–3 weeks prior to imaging studies.

### 2.3. Antibody Conjugation and Administration

The humanized anti-CEA hT84.66-M5A monoclonal antibody, established by Yazaki et al., was used for tumor labeling in this study [10]. M5A was conjugated to the near-infrared (NIR) dye IRDye800CW NHS Ester (LI-COR Biosciences, Lincoln, NE, USA) as per the manufacturer’s instructions [20]. Conjugated antibodies were stored at 4 °C. For its administration to orthotopic mouse models, 50 µg of M5A-IR800 was diluted in PBS for a total injection volume of 100 µL and administered via the tail vein. Imaging was performed after 72 h.

### 2.4. Imaging

Two weeks after the establishment of the orthotopic mouse models, non-invasive imaging of luciferase–luciferin was performed to monitor tumor size using the bioluminescence channel of the Pearl Trilogy Small Animal Imaging System (LI-COR, Lincoln, NE, USA). After receiving appropriate anesthesia, mice received 4.5 mg (1.5 mg per 10 g of body weight) of D-Luciferin via intraperitoneal injection (Goldbio, St. Louis, MO, USA). Based on tumor size, six of the mice received M5A-IR800 on the day of luciferase–luciferin imaging. The remaining three mice waited an additional week to allow greater tumor growth prior to receiving M5A-IR800. Seventy-two hours after the injection of M5A-IR800, mice again received a weight-based injection of D-luciferin prior to euthanasia and laparotomy to allow intra-abdominal imaging. Near-infrared (NIR) and bioluminescence imaging were performed with the Pearl Trilogy Small Animal Imaging System. NIR imaging was also performed using the Stryker 1688 Advanced Imaging Modalities (AIM) 4k platform 10 mm laparoscope (Stryker Corp, Kalamazoo, MI, USA) and the FLARE imaging system (Curadel, Natick, MA, USA). The mean fluorescence intensity (mFI) of the tumor and the normal liver in the 800 nm channel of the Pearl Trilogy Small Animal Imaging System were measured in each mouse and the tumor-to-liver ratio (TLR) was calculated. After imaging the metastatic tumor, a necropsy was performed. The metastatic tumor, liver, spleen, stomach, pancreas, cecum, kidney, lung, and ear were collected and imaged with the Pearl Trilogy Small Animal Imaging System to obtain fluorescence biodistribution data.

### 2.5. Quantification of Signal Co-Localization

Within the Pearl Trilogy Small Animal Imaging System, analysis circles were drawn around areas of fluorescence signals to calculate the total area of M5A-IR800 labeling for each mouse. To calculate the area for the ‘true tumor’, analysis circles were drawn using the combined bright light and luciferase–luciferin images. Both image layers were used, as the large tumors often had patchy labeling of luciferase–luciferin (thought possibly due to tumor necrosis affecting processing of luciferin) while the small tumors were nearly imperceptible upon bright light exposure alone. A ratio of the areas for the M5A-IR800 and the ‘true tumor’ were then calculated.

### 2.6. Immunohistochemistry and Immunofluorescence

Tumor samples were removed en bloc with surrounding tissue at the time of mouse necropsy. Samples were fixed in formalin for at least 72 h prior to being embedded in paraffin and sectioned. Hematoxylin and eosin staining was performed per standard protocols. Immunohistochemistry was performed using the humanized anti-CEA hT84.66-M5A antibody at a dilution of 1:1000 with secondary goat anti-human horseradish peroxidase (Southern Biotech, Birmingham, AL, USA) at a dilution of 1:200. Horseradish peroxidase was visualized by a diaminobenzidine (DAB) chromogenic reaction. Immunofluorescence was performed per a standard protocol using Opal fluorophores (Opal 690, PerkinElmer Inc., Waltham, MA, USA) for CEA and then counterstained with 4,6-diamidino-2-phenylindole dihydrochloride (DAPI) (Sigma-Aldrich, St. Louis, MO, USA) to visualize the nuclei of cells.

### 2.7. Statistical Analysis

Statistical analysis was performed using R software version 2024.04.1+748 (Free Software Foundation, Boston, MA, USA). The mFI of the tumors and normal liver were both normally distributed, thus, a student’s *t*-test was performed. A *p*-value of <0.05 was used as a predetermined cutoff for statistical significance.

## 3. Results

### 3.1. Non-Invasive and Intra-Abdominal Imaging of CRLMs Using the Pearl Trilogy Small Animal Imaging System Demonstrating Co-Localization of the Luciferase-Luciferin and M5A-IR800 Signals

**Figure 1:**
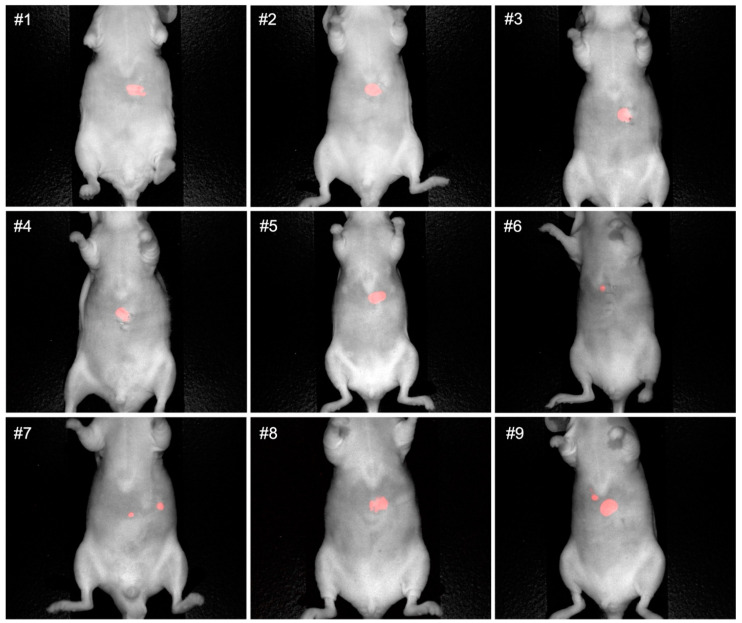
Non-invasive luciferase–luciferin imaging of CRLMs in an orthotopic nude mouse model derived from LS174T–luciferase cells with the Pearl Trilogy Small Animal Imaging System two weeks after tumor implantation.

**Figure 2:**
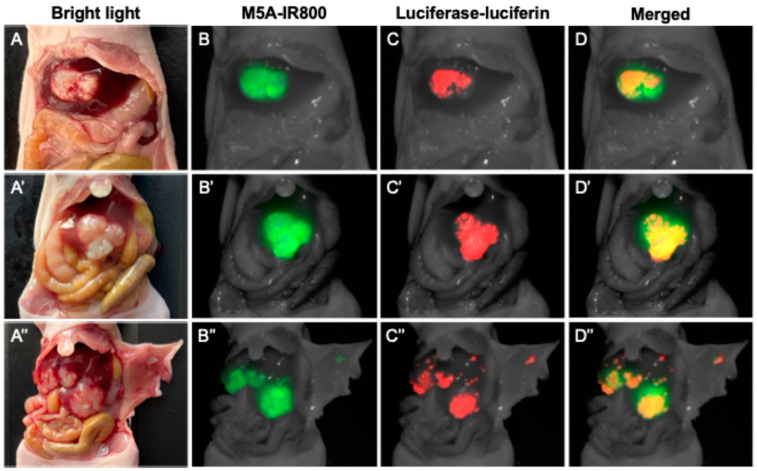
Intra-abdominal imaging in nude mice of CRLMs derived from LS174T–luciferase cells with the Pearl Trilogy Small Animal Imaging System 72 h after M5A-IR800 injection in mouse 6, 8, and 9. (**A**–**A″**) Bright light images of CRLMs. (**B**–**B″**) Near-infrared imaging of M5A-IR800. (**C**–**C″**) Bioluminescence imaging of luciferase–luciferin. (**D**–**D″**) Co-localization of M5A-IR800 and luciferase–luciferin in all CRLMs and peritoneal metastases.

**Figure 3:**
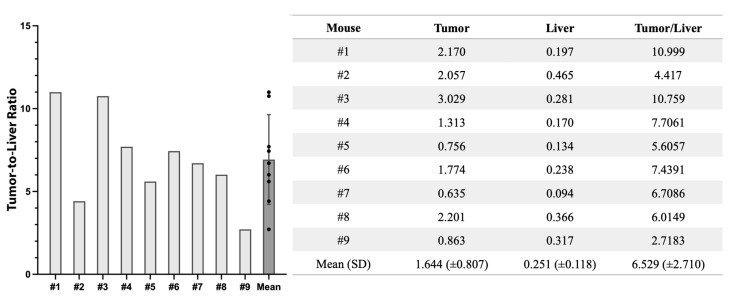
Tumor-to-liver ratios at 800 nm fluorescence with the Pearl Trilogy Small Animal Imaging System 72 h after M5A-IR800 injection in nude mice with CRLMs derived from LS174T–luciferase cells. SD, standard deviation.

Non-invasive luciferase–luciferin imaging clearly visualized CRLMs in all mice (Figure 1). Intra-abdominal luciferase–luciferin imaging and NIR imaging of M5A-IR800 were performed and showed excellent co-localization of the tumors (Figure 2). In two of the orthotopic models with advanced tumors (mouse 8 and 9), peritoneal metastases and multiple liver metastases developed (Figure 2A′,A″). The average mFI of tumors was 1.644, and the average mFI of the normal liver was 0.251 (*p*-value = 0.0008). The tumor-to-liver ratios (TLRs) ranged from 2.72 to 10.99 with an average TLR of 6.52 (standard deviation = 2.71) (Figure 3).

### 3.2. Quantification of Signal Overlap

**Figure 4:**
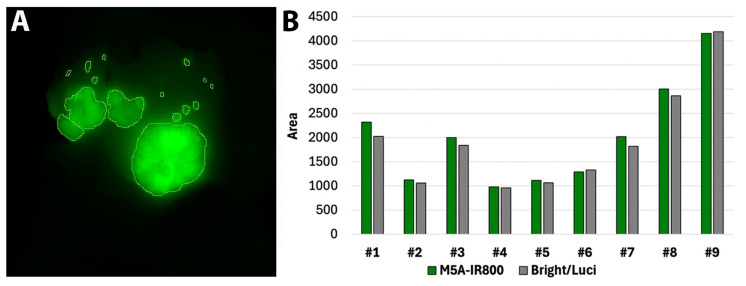
M5A-IR800 accurately labels CRLMs. (**A**) Calculation of M5A-IR800 signal with the Pearl Trilogy Small Animal Imaging System. (**B**) Quantification of signal overlap between M5A-IR800 and true tumor as determined by bright light and luciferase–luciferin signals.

Within the Pearl Trilogy Small Animal Imaging System, analysis circles were drawn around areas of fluorescence signal to calculate the total area of M5A-IR800 labeling for each mouse (Figure 4A). The same was performed using the combined bright light and luciferase–luciferin images to determine the area for the ‘true tumor’. The comparative values for each mouse are represented in Figure 4B. On average, M5A-IR800 labelling encompassed 1.05 times more area than the bright light and luciferase–luciferin (range 0.97–1.14) suggesting excellent co-localization.

### 3.3. Targeting of Metastases with M5A-IR800 Imaged with the Stryker 1688 Laparoscope

**Figure 5:**
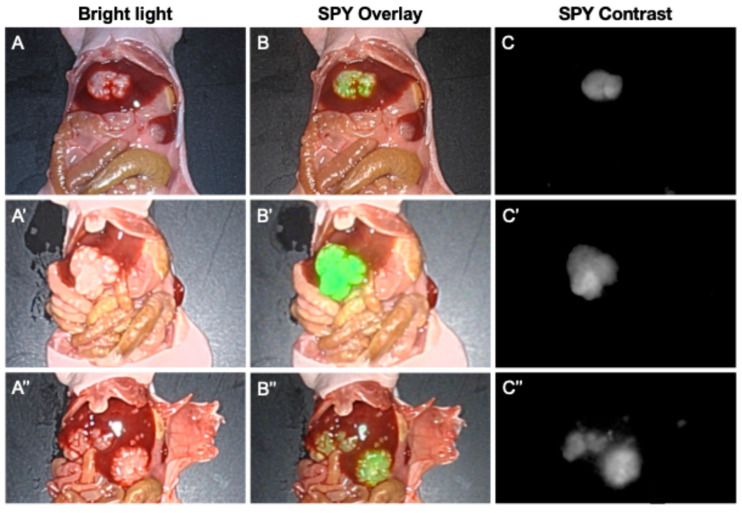
Intra-abdominal imaging of orthotopic nude mouse models (#6, 8, and 9) of CLRMs derived from LS174T cells with a Stryker 1688 Imaging System 72 h after M5A-IR800 injection. (**A**–**A″**) White light images showing liver metastases. (**B**–**B″**) SPY Overlay images with M5A-IR800 labeling of CRLMs in green. (**C**–**C″**) SPY contrast images with bright M5A-IR800 labeling of CRLMs.

The efficacy of M5A-IR800 in targeting and labeling CRLMs was tested using the Stryker 1688 laparoscope, a commercially available clinical imaging device. Bright contrast was seen compared to the surrounding tissues with both the SPY color overlay and SPY black and white contrast modes of the Stryker 1688 (Figure 5). Metastatic tumors as small as 1 mm were clearly detected.

### 3.4. M5A-IR800 Targeting of CRLMs Imaged with the FLARE Imaging System

**Figure 6:**
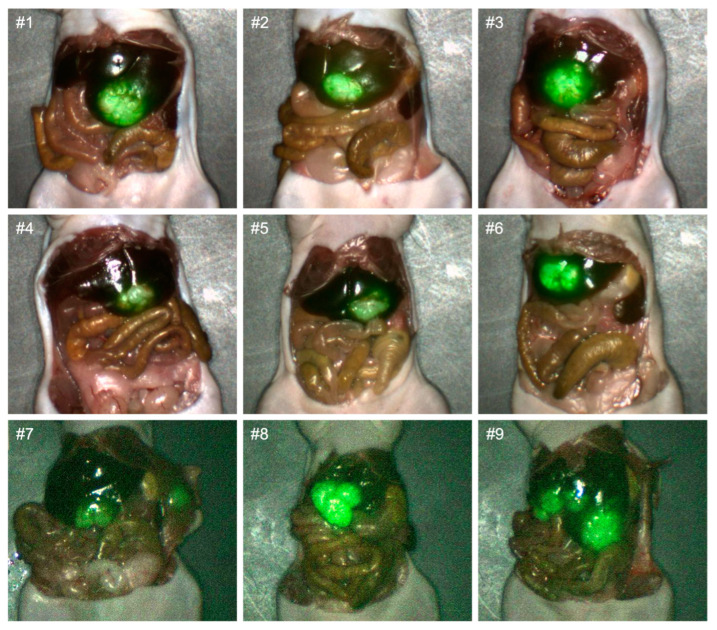
Imaging of CLRMs derived from LS174T cells with the FLARE Imaging System 72 h after M5A-IR800 injection.

The bright labeling of CLRMs was seen with M5A-IR800 compared to the surrounding liver and other intra-abdominal organs with the FLARE imaging system (Figure 6).

### 3.5. Fluorescence Biodistribution of M5A-IR800

**Figure 7:**
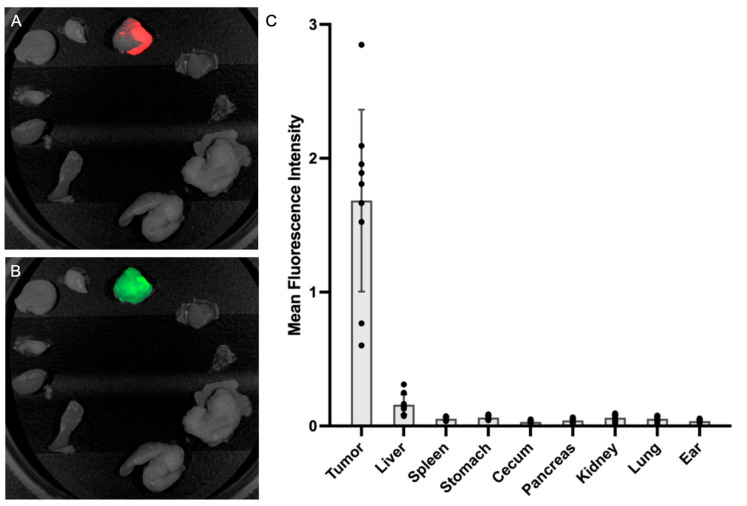
The fluorescence biodistribution of M5A-IR800 in the LS174T–luciferase CRLMs and various organs with the Pearl Trilogy Small Animal Imaging System. (**A**) A representative image of luciferase–luciferin signals confined to known CRLM tumors, confirming the absence of cancer cells in normal-appearing organs. (**B**) Minimal fluorescence of M5A-IR800 in any organs except the tumors. (**C**) Mean fluorescence intensity (mFI) values of M5A-IR800 in tumors and organs. Tumor = 1.684 (SD = 0.679), liver = 0.160 (SD = 0.075), spleen = 0.055 (SD = 0.012), stomach = 0.063 (SD = 0.015), cecum = 0.030 (SD = 0.008), pancreas = 0.042 (SD = 0.012), kidney = 0.061 (SD = 0.024), lung = 0.055 (SD = 0.018), and ear = 0.037 (SD = 0.009). Error bars: standard deviation. Dots: mFI for individual mice.

After intra-abdominal imaging, necropsy was completed and fluorescence biodistribution was collected using the Pearl Trilogy Small Animal Imaging System. The absence of tumor deposits within normal-appearing organs was confirmed using luciferase–luciferin imaging (Figure 7A). The fluorescence signal of M5A-IR800 was also obtained for each organ. A minimal signal was seen in all, organs except for the metastatic tumors (Figure 7B). The average values for all mice are represented in Figure 7C.

### 3.6. Immunohistochemistry and Immunofluorescence

**Figure 8:**
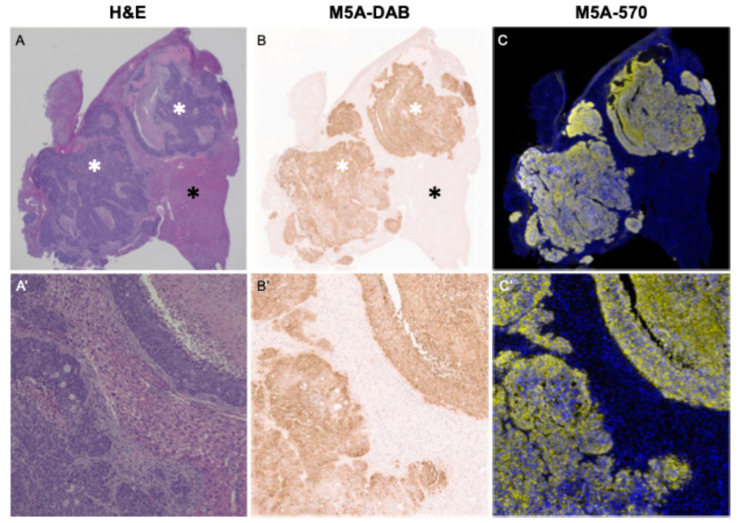
Strong CEA staining of CRLM adenocarcinoma cells using the M5A antibody. (**A**) H&E staining observed at 2× magnification of the CRLM adenocarcinoma denoted by a white asterisk and normal liver tissue by a black asterisk. (**A′**) 20× magnification of the H&E stained CRLMs. (**B**) Immunohistochemical staining of M5A at 2× magnification. (**B′**) 20× magnification of M5A staining. (**C**) M5A immunofluorescence staining (yellow) using Opal 570 at 2× magnification with DAPI staining (blue), showing strong M5A staining within the adenocarcinoma cells. (**C′**) 20× magnification of Opal 570 and DAPI staining.

Hematoxylin and eosin (H&E) staining showed well-to-moderately differentiated adenocarcinoma (Figure 8A). Immunohistochemical and immunofluorescence CEA staining using the M5A antibody were strongly positive and confined to areas of CRLM adenocarcinoma. These results confirm the specific targeting of the M5A-IR800 antibody–dye conjugate (Figure 8B,C).

## 4. Discussion

The present study demonstrated the accuracy of CRLM targeting with an anti-CEA antibody, M5A-IR800, by its co-localization with luciferase–luciferin signals of the cancer cells in an orthotopic CRLM mouse model. All images demonstrated high resolution tumor labeling, even in tumors smaller than 1 mm, with both the Pearl and the Stryker imaging systems.

Luciferase is a class of oxidative enzymes that produces bioluminescence by emitting a photon. It has been widely used in biotechnology, especially for the imaging and detection of metastatic cancer in preclinical studies [21]. Evaluating the development of metastases in animal models has been challenging in the past. However, employing bioluminescence imaging with luciferase-labeled cancer cells allows for the easy detection and monitoring of cancer metastases non-invasively and in real time [22]. Luciferase–luciferin imaging has been used for studies of metastasis and tumor biology in many types of cancer [23,24] and has been evaluated for its efficacy compared to fluorescence imaging [25].

In the present study, luciferase–luciferin images showed a clearly delineated tumor edge and acted as the true positive signal against which M5A-IR800 targeting was compared. Fluorescence images of M5A-IR800, acquired in the NIR 800 nm channel, targeted the CRLM tumors and co-localized with the bioluminescence signal, demonstrating the accuracy of the probe. The use of this dual imaging modality with bioluminescence and NIR fluorescence has the potential to change the standard by which candidate probe testing for use in FGS is conducted.

Tumor-targeted, fluorescence-guided surgery (FGS) has emerged as a promising technique for the intraoperative detection and resection of many solid tumors [26,27,28,29,30,31]. Numerous studies evaluating target molecules have been reported [32,33,34,35,36,37] and clinical trials on various cancers are actively ongoing [38]. Among the numerous tumor-specific targets, CEA is one of the most well studied, as it has shown efficacy in terms of labeling numerous gastrointestinal solid tumors including colorectal cancer [39]. However, fluorescence signals may accumulate in the liver or bladder due to their metabolism in the early period after injection. Fluorescence accumulation in the liver may interfere with the visualization of the tumor, which becomes challenging in the case of primary liver tumors or CRLMs [17]. In a pilot clinical trial applying M5A for CEA-expressing tumors using PET imaging, false negative cases were seen and attributed to high signals in the normal liver [40]. Turner et al. reported the efficacy of using 75 micrograms of PEGylated M5A-IR800 with imaging 96 h after injection to minimize hepatic accumulation [18]. In the present study, by using a lower dose of M5A-IR800 (50 micrograms), we were able to achieve a lower background liver signal despite imaging at an earlier timepoint. This was true with all three imaging platforms: the Pearl system, the Stryker 1688 platform, and the FLARE imaging system.

In the present study, we validated M5A-IR800 for the selective targeting of liver metastases with the co-localization of bioluminescence and the fluorescence signal of M5A-IR800 using the Stryker 1688 Advanced Imaging Modalities 4K Platform (a commercially available clinical imaging device). Although the signal intensity of each mouse was variable, the SPY Overlay mode clearly demonstrated fluorescence signals throughout the tumor without background liver signals. The contrast between tumor and normal liver was even more striking in the SPY contrast mode, though the resolution compared to the Pearl small animal imaging system was slightly lower. Particularly, in mouse #9, which had advanced liver metastases as small as 1 mm, each of the metastases were well visualized in the SPY contrast mode (Figure 5C″).

There are several limitations of this study. The first is the use of a single human colorectal cancer cell line. LS174T was selected as it has high levels of CEA expression compared to other cell lines [41]. LS174T is widely used for colorectal cancer research as the majority of human colorectal cancers, particularly those with metastatic disease, express CEA. Additionally, we have previously demonstrated the ability of M5A-IR800 to target a second colorectal cancer cell line, HT-29 [20]. However, the primary objective of this report was to validate prior work with M5A in colorectal cancer and to potentially propose a new standard by which the preclinical testing of FGS probes can be conducted.

Second, FGS may not be effective for the very early stages of CRLM development. However, tumor-targeted FGS can still increase the detection rate of small lesions that may be missed by the naked eye or by preoperative imaging. Third, the progression of tumors in orthotopic animal models likely does not fully reflect every stage of CRLM development especially the early stage, as mentioned above. Despite this model relying on the direct implantation of tumors into the liver, numerous small liver metastases were able to be generated in the models as seen in Figure 2.

Although the technology of tumor-specific FGS is developing rapidly, the validation and standardization of protocols are needed for its clinical translation into surgical practice and, consequently, to improve the oncologic benefits of this strategy. The present study demonstrates for the first time the power of co-localizing a signal from the liver metastases, in this case luciferase–luciferin, and the signal from the antibody probe, in this case M5A-IR800, to demonstrate the accuracy of the antibody’s detection of liver metastases.

## 5. Conclusions

M5A-IR800 co-localized with luciferase-expressing CRLMs in an orthotopic mouse model, which confirmed the accuracy of M5A-IR800 metastatic targeting and demonstrated its promising clinical potential for use in fluorescence-guided surgery.

## Data Availability

The original contributions presented in the study are included in the article; further inquiries can be directed to the corresponding author.
